# Efficient eco-friendly inverted quantum dot sensitized solar cells†
†Electronic supplementary information (ESI) available: TEM images of QDs, XPS spectra, UV-vis and PL spectra of the sensitized electrodes, details about photophysical characterization and IPCE spectra interpretation. See DOI: 10.1039/c5ta06769c



**DOI:** 10.1039/c5ta06769c

**Published:** 2015-12-01

**Authors:** Jinhyung Park, Muhammad T. Sajjad, Pierre-Henri Jouneau, Arvydas Ruseckas, Jérôme Faure-Vincent, Ifor D. W. Samuel, Peter Reiss, Dmitry Aldakov

**Affiliations:** a Univ. Grenoble Alpes , INAC-SPRAM , F-38000 Grenoble , France . Email: dmitry.aldakov@cea.fr; b CNRS , INAC-SPRAM , F-38000 Grenoble , France; c CEA , INAC-SPRAM , F-38000 Grenoble , France; d Organic Semiconductor Centre , SUPA , School of Physics and Astronomy , University of St Andrews , North Haugh, St Andrews , Fife , UK . Email: idws@st-andrews.ac.uk; e CEA , INAC-SP2M , LEMMA , F-38000 Grenoble , France

## Abstract

Recent progress in quantum dot (QD) sensitized solar cells has demonstrated the possibility of low-cost and efficient photovoltaics. However, the standard device structure based on n-type materials often suffers from slow hole injection rate, which may lead to unbalanced charge transport. We have fabricated efficient p-type (inverted) QD sensitized cells, which combine the advantages of conventional QD cells with p-type dye sensitized configurations. Moreover, p-type QD sensitized cells can be used in highly promising tandem configurations with n-type ones. QDs without toxic Cd and Pb elements and with improved absorption and stability were successfully deposited onto mesoporous NiO electrode showing good coverage and penetration according to morphological analysis. Detailed photophysical charge transfer studies showed that high hole injection rates (10^8^ s^–1^) observed in such systems are comparable with electron injection in conventional n-type QD assemblies. Inverted solar cells fabricated with various QDs demonstrate excellent power conversion efficiencies of up to 1.25%, which is 4 times higher than the best values for previous inverted QD sensitized cells. Attempts to passivate the surface of the QDs show that traditional methods of reduction of recombination in the QD sensitized cells are not applicable to the inverted architectures.

## Abstract 2

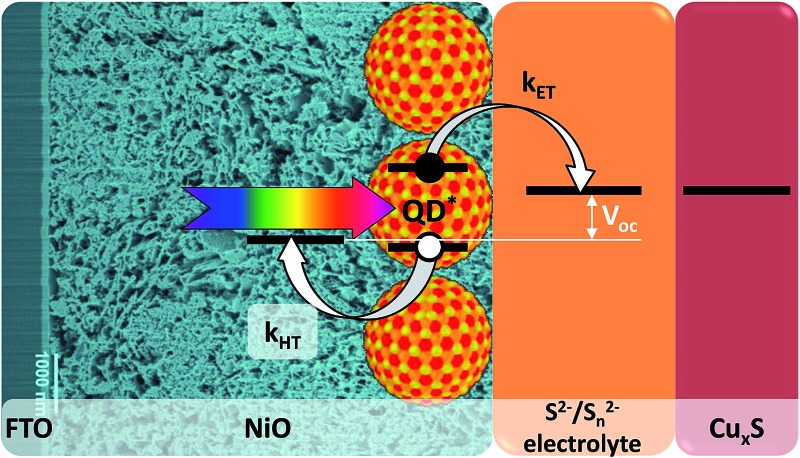
Inverted quantum dot sensitized solar cells using non-toxic CuInS_*x*_Se_2–*x*_ nanocrystals deposited on mesoporous NiO demonstrate high hole injection rates of 10^–8^ s^–1^ and record efficiencies of 1.25%..

## Introduction

Semiconductor quantum dot (QD) sensitized cells (QDSSCs) initially inspired by the concept of dye-sensitized solar cells (DSSC) have evolved into a separate rapidly growing field due to the unique properties of QDs, such as high absorption, easy tunability of the energy levels and processing from colloidal solutions. In recent years significant progress has been achieved in various aspects of QDSSCs: their power conversion efficiency has recently reached 8.21%,^1^ while the fabrication has been greatly simplified,^2,3^ and stability issues have been improved.^4–6^ Several points have been identified as potential routes for further improvement such as proper energy alignment,^7^ strong electronic coupling between all the components,^8^ QD surface passivation, and higher loading of QDs. Most of these issues can be addressed by designing QDs with tailored band gaps and optimized organic or inorganic surface passivation. However recently, several groups have concluded that the key factor limiting the performance of QDSSCs is hole transfer from QDs to the redox couple.^9–11^ Even though it can be improved using classical strategies such as inorganic shell growth to reduce the charge recombination, the rate of hole transfer from the excited QD to redox couple will remain inherently slower and less efficient than electron transfer to a nanostructured semiconductor scaffold. This leads to unbalanced charge transport in the cell and so, alternative approaches to the design of QDSSCs should be explored.

Similar shortcomings observed in DSSCs have led to the emergence of the cells of second generation based on the principle of hole injection from excited organic dyes to nanostructured p-type semiconductors, such as NiO.^12,13^ While being much less investigated than conventional Grätzel cells, p-DSSCs attract increasing attention over last years and due to a series of recent developments a record efficiency of 2.51% has been achieved.^14^ Moreover, probably the most important and promising application of p-type sensitized solar cells is their combination with n-type ones in a tandem configuration.^13^ Indeed, combining two absorbers in the photoanode and the photocathode can allow overcoming the Shockley–Queisser limit. Recently, very promising first examples of the use of such tandem architectures have been demonstrated for dye sensitized solar cells^15–17^ and water splitting.^18^ Among the reasons for the generally lower efficiency of these cells compared to n-DSSCs the most critical point is fast recombination of holes injected into the p-type semiconductor with the reduced dye.^13,19,20^ It is also worth mentioning in this context the progress obtained last year in the field of hybrid perovskite solar cells using NiO as p-type electrode in an inverted cell structure.^21–26^ An impressive efficiency value of 17.3% was reached very recently for nanostructured NiO obtained *via* pulsed laser deposition.^27^ At the same time, the field of hybrid perovskite photovoltaics has its own problems to be solved such as for example the presence of toxic lead in soluble form and the low operational stability and it is therefore important to continue to develop alternative solutions.

**Fig. 1:**
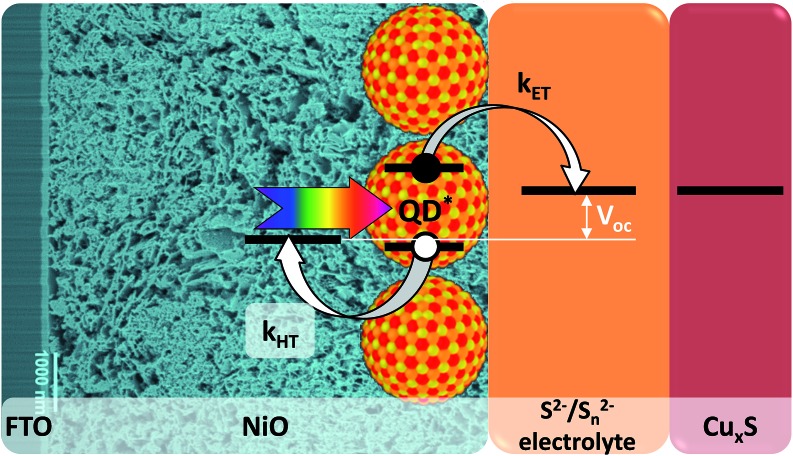
Working principle of a p-type QDSSC.

By combining the advantages of n-QDSSCs and p-DSSCs for the design of an inverted QDSSC (p-QDSSC) one can overcome their inherent weak points. Such a cell can benefit from the easy tuning of optoelectronic properties of the QDs in order to adjust the energy levels in the system and optimize the hole injection while the whole arsenal of QD surface chemistry methods can be used to reduce the recombination of the separated charges. In such cell after the light absorption the QDs inject a hole into a nanostructured p-type wide band gap semiconductor, while the electron is regenerated by the redox electrolyte and recovered on a counter-electrode (Fig. 1). Even though the concept of inverted cells sensitized by inorganic nanomaterials is very appealing, most attempts to fabricate them made in recent years showed very low efficiencies. Typically, because of problems with energy alignment and/or charge recombination, previous research efforts were generally limited to charge transfer^28–30^ and photoelectrochemical studies^31–33^ while the photovoltaic efficiency was very low or not reported^34–36^ despite relatively decent incident-photon-to-electron conversion efficiency (IPCE) values reaching in some cases 30%.^33^ To the best of our knowledge, for solar cells using p-QDSSCs architecture the highest efficiency reported so far is 0.35%.^37^ Generally, the layer of inorganic sensitizer is deposited on the p-type semiconductor by *in situ* fabrication using chemical bath deposition (CBD),^28^ successive ionic layer adsorption and deposition (SILAR),^33,37^ electrodeposition^34^ or spray pyrolysis.^35^ None of these methods gives control over the surface states or over the size distribution (and thus energy levels) of the nanometric coatings, both of which are extremely important for efficient cell functioning. Barcelo *et al.* compared in detail NiO/CdS assemblies obtained by different QD deposition techniques and concluded that the charge injection efficiency follows the order: directly adsorbed colloidal QDs > colloidal QDs deposited *via* linker > SILAR.^31^ This study demonstrates that ex situ synthesized colloidal QDs are better adapted for p-QDSSCs because of the higher degree of control of their physical properties.

In most previous studies on QDSSCs the inorganic sensitizers were composed of cadmium chalcogenides (CdS or CdSe). An obvious disadvantage of this type of sensitizers is the toxicity of cadmium compounds whose use should be avoided. Recently, a series of important research works have reported the successful use of colloidal ternary or quaternary QDs for solar cells,^6,38–42^ such as CuInS_2_, CuInSe_2_, AgInS_2_ which are non-toxic as they do not contain heavy metals, have high absorption coefficients, long photoluminescence lifetimes and band gaps (1–1.5 eV) adapted for efficient sunlight harvesting.^43^ Moreover, due to crystalline flexibility and defect tolerance such QDs offer alternative band gap tuning strategies in addition to the classical size control: a mixture of the chalcogenide atoms (S^2–^, Se^2–^) and introduction of new cations (Zn^2+^, Ga^3+^) into the ternary crystalline structure allows varying the band gap in a wide range.^44–46^ For example, upon introduction of selenium into CuInS_2_ QDs the band gap decreases thus extending the absorption spectrum and allowing to harvest more infrared photons of the sunlight.^6^ Another technique that has proved highly beneficial for photovoltaic applications is QD surface passivation by inorganic materials. To give an example, reduced recombination losses were observed in QDSSCs sensitized with CuInS_2_ QDs when using surface cation exchange with Zn^2+^.^47^

In the present paper we report on the development of p-type QDSSCs based on mesoporous NiO sensitized with non-toxic ternary quantum dots of CuInS_2_ and CuInS_*x*_Se_2–*x*_ with various ligands and surface passivation. The morphology of the obtained assemblies of NiO/QDs, studied in detail by scanning transmission electron spectroscopy (STEM) with High Angle Annular Dark Field (HAADF) detector and Energy Dispersive X-ray spectrometer (EDX), shows excellent penetration of the QDs into the mesoporous NiO layer, which can enhance the QD loading and lead to better light harvesting and charge transfer properties. Transient photoluminescence spectroscopy was used to study hole transfer processes in the cells and high hole injection rates from QDs to NiO of 10^8^ s^–1^ were observed, which is comparable to the electron injection rates in analogous n-type TiO_2_/CuInS_2_ QDs solar cells. Inverted cells fabricated using such assemblies yielded high photoconversion efficiencies of up to 1.25%, *i.e.* around 4 times more than the highest value reported in this field. Our approach offers a pathway to efficient inverted QD solar cells and paves the way to tandem sensitized cells.

## Results and discussion

### Morphological studies

**Fig. 2:**
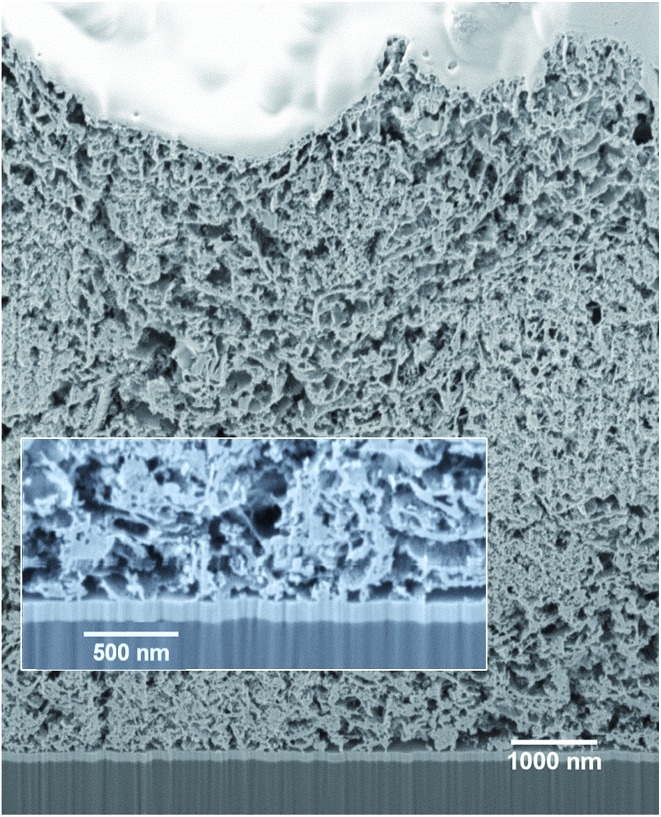
SEM images of FIB cross-section of NiO mesoporous substrate at different magnifications.

The mesoporous nickel oxide electrodes used in this work were prepared from a commercial paste by using an optimized procedure (see Experimental section). They demonstrate high porosity and interconnectivity of the pores on the Focused Ion Beam (FIB) cross-section SEM image (Fig. 2). In addition, a high resolution HAADF-STEM image of the bottom of the mesoporous layer shows the high contact surface between the NiO and FTO, which is essential to avoid delamination and to achieve high efficiency of hole injection into the electrode. Estimation of the porosity of mesoporous NiO (fraction of pores in total volume) by segmentation of the FIB-SEM image and density ratios gave similar high values of 0.85 and 0.8, respectively. X-ray photoelectron spectroscopy (XPS) analysis carried out on the mesoporous NiO films shows its purity with only trace amounts of adventitious carbon and complete absence of metallic Ni^0^ contrary to most of the NiO reported in literature (Fig. S3†).^48^

Transmission electron microscopy (TEM) allows determining the shape and average size of the CuInS_*x*_Se_2–*x*_ QDs as tetrahedrons with a height of 4.9 ± 0.3 nm and an edge length of 6.4 ± 0.2 nm (see Fig. S1†). Selected area electron diffraction (SAED) has been used for the determination of the crystal structure lattice parameters of the obtained QDs. While it is generally difficult to differentiate between the cubic zinc blende and the tetragonal chalcopyrite phase in these systems, the observed 2*a*/*c* ratio of 1.016 indicates that the latter is predominant in the present case (Fig. S1†). The band gap of CuInS_*x*_Se_2–*x*_:Zn^2+^ QDs calculated from electrochemical measurements was about 1.9 eV, which means that the QDs are in the quantum confinement regime (the band gap values for bulk CuInS_2_ and CuInSe_2_ are 1.5 and 1.15 eV, respectively). It is corroborated by the fact that the size of such alloyed QDs is above the Bohr radius of CuInS_2_ but below the one for CuInSe_2_ (4.1 and 10.6 nm, respectively).

After the deposition of the QDs on the electrode XPS analysis shows unambiguous presence of all the elements of CuInS_*x*_Se_2–*x*_:Zn^2+^, moreover the initial peaks of NiO are intact confirming that the deposition process does not alter the chemical composition of NiO (Fig. S11†).

**Fig. 3:**
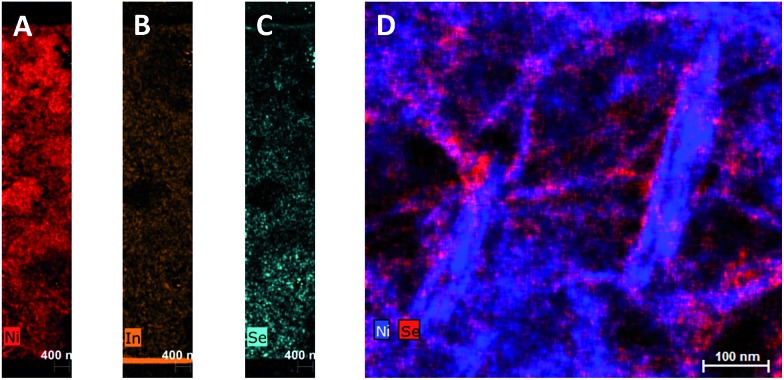
STEM EDX cartography of a slice of NiO/CuInS_*x*_Se_2–*x*_:Zn^2+^ film fabricated by FIB. (A–C): Full substrate thickness slices; (D): high resolution image with Ni and Se elements.

To investigate the penetration of the QDs into the mesoporous layer of NiO after the solution deposition, we have performed FIB-assisted cross-section of the sensitized films followed by HAADF-STEM studies coupled with EDX analysis. On the image of NiO/QD layer it is problematic to distinguish individual QDs because the size is comparable to the morphological features of the NiO itself (see Fig. S5†). At the same time, an image containing chemical information allows to highlight QDs. In the case of CuInS_*x*_Se_2–*x*_, we have chosen indium and selenium as EDX elemental markers because their peaks are well resolved and can be easily separated from the contributions of nickel and oxygen originating from NiO, whereas copper and sulfur can be present in the sample support (copper TEM grid) and/or top conducting coating. STEM EDX microscopy reveals that the QDs fill homogeneously all the depth of the NiO layer down to the fluorine-doped tin oxide (FTO) layer (Fig. 3A–C and S10†). Higher resolution microscopy allows obtaining a detailed image of the QDs deposited on NiO showing that the former are indeed attached to the walls leaving the interior volume of the pores void, which is beneficial for the subsequent complete solvent pore filling, and its good contact with the excited QDs for charge extraction (Fig. 3D). Moreover, we can conclude that the QD loading on mesoporous NiO is high judging from the respective Se intensity superimposed on Ni one on high resolution images.

### Photophysical studies

In order to elucidate charge transfer dynamics in the systems of NiO with QDs steady-state and time-resolved photophysical studies of the QD films on glass and on NiO electrodes have been performed. First, photoluminescence quantum yields (PL QYs) of QD have been measured on glass, where the PL intensity is defined exclusively by intrinsic defects of the QDs. PL QY values obtained for QDs on glass (1–2%) are lower compared to 20% reported for similar QDs in solution.^6^ This behavior is typical for solid films of close-packed QDs, in which Förster resonance energy transfer and/or excited charge-carrier transfer processes can take place^49^ (Table 1). Films of CuInS_*x*_Se_2–*x*_ exhibit slightly lower PL QY with respect to QDs without selenium, which has been previously observed in the case of solutions in previous reports.^47^ After the deposition onto the mesoporous NiO electrodes the QD PL efficiency considerably decreases, which is manifested by a dramatic drop in the measured QY (5 to 10 times lower than QD on glass). This drop is interpreted in terms of NiO acting as an efficient quencher for the excited QDs in direct contact. In addition to typical non-radiative recombination pathways occurring in excited QD solids such as surface defects (dangling bonds) or deep traps (element vacancies or interstitials), proximity of electronic band structure of NiO provides a new de-excitation channel, *i.e.* hole transfer from the valence band (VB) of a QD to the VB of NiO. Provided that this alternative process is efficient and fast compared to the radiative decay, it can further decrease the PL QY, which is indeed observed in the present work.

**Table 1:** Photophysical properties of QDs deposited on glass and on NiO

QDs	Substrate	PL QY [%]	*k*_HT_ [s^–1^]
CuInS_2_:Zn^2+^	Glass	2.0	5.4 × 10^7^
	NiO	0.2	
CuInS_2_:Cd^2+^	Glass	1.1	3.9 × 10^7^
	NiO	0.2	
CuInS_2_Se_2–*x*_:Zn^2+^	Glass	0.9	8.2 × 10^8^
	NiO	0.1	
CuInS_2_Se_2–*x*_:Cd^2+^	Glass	1.0	5.1 × 10^8^
	NiO	0.1	

To unravel the interplay of all the processes emerging after the excitation of QDs in contact with NiO from a timescale viewpoint, time-resolved photoluminescence studies of NiO/QD films have been performed. First, QD films on glass were excited and their PL decay measured. In agreement with previous studies, a triexponential model appears to be optimal to fit the decay, each exponential corresponding to one of three components: radiative decay involving surface states (shortest decay time on the order of 1 ns), closest and next-to-closest donor acceptor pair recombination (typical decay times of 10 ns and 100 ns, respectively).^50,51^ Therefore, unlike classical CdS or CdSe QD systems, the fitting of the CuInS_2_ and CuInS_*x*_Se_2–*x*_ PL decays results in three distinct lifetimes. These could in principle be averaged but taking into account the different underlying de-excitation processes it appears judicious to avoid the use of average lifetimes (details about fitting parameters are given in Table S2, ESI†). Upon the deposition of QDs on NiO, significant shortening of all three components of the PL decay was observed compared to those of QD films on glass as well as a dependence on the QDs composition. For the QDs in this study, the long-lived component decreased by a factor of 5 to 12. Similar substantial reduction of the lifetime has been previously demonstrated in the case of individual CdSe QDs deposited on NiO.^30^ The interpretation of this observation is based on the same grounds as the decrease of the QY, *i.e.* appearance of an alternative fast non-radiative decay pathway related to the hole transfer (HT) from the excited QD to NiO due to the favorable position of the energy levels of both components.

**Fig. 4:**
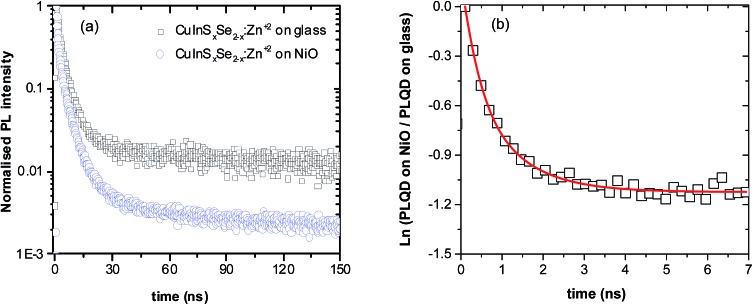
(a) PL decay profiles of CuInS_*x*_Se_2–*x*_:Zn^2+^ QDs on glass and on NiO. (b) Natural logarithms of ratio of PL of QD on NiO and PL of QD on glass. The red line is a fit used to calculate time dependent hole transfer rate.

One of the indicators of the efficiency of this hole transfer is an apparent HT constant, *k*_HT_, which is typically calculated using:1
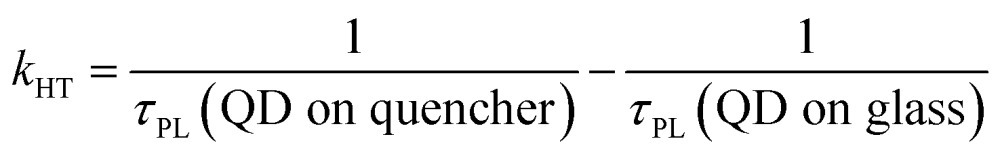
where *τ* is a decay lifetime. While being well adapted for systems with a simple monoexponential decay, it fails to provide physically meaningful information of the charge transfer in the case of ternary QDs used in this work which do not have monoexponential decay. Instead we take the natural logarithm of the ratio of decay on NiO to the decay on glass. The resulting graph is shown in Fig. 4b and its slope is equal to the rate of quenching.^42,52^ The equivalent graphs for CuInS_*x*_Se_2–*x*_:Cd^2+^, CuInS_2_:Zn^2+^ and CuInS_2_:Cd^2+^ are given in ESI.† We fit the ln(PL ratio) to a sum of three-exponentials before differentiation and obtain the hole transfer rate from2
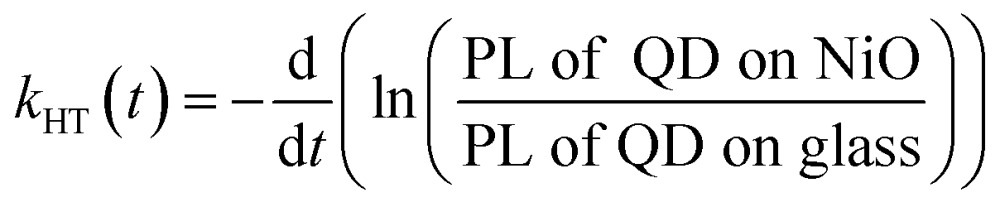



**Fig. 5:**
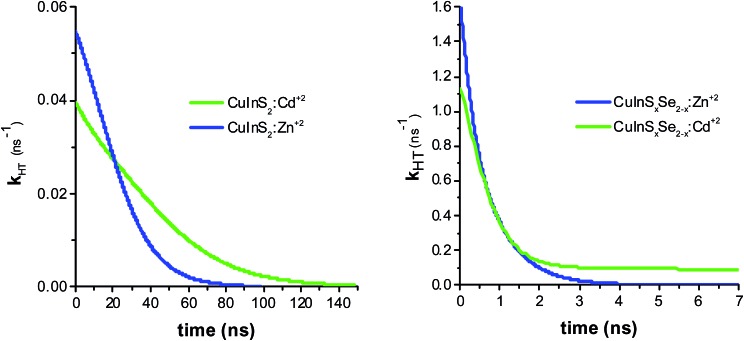
Hole transfer rate determined by taking the derivative of PL ratios for CuInS_2_:Zn^2+^ and CuInS_2_:Cd^2+^ (left panel), and CuInS_*x*_Se_2–*x*_:Zn^2+^ and CuInS_*x*_Se_2–*x*_:Cd^2+^ (right panel).

The resulting hole transfer rates for the four materials studied are shown in Fig. 5. The key point is that quenching rate (hole transfer rate) is very strongly time-dependent.

For comparison with other studies, we estimated the average hole transfer rate by integrating the time dependent rate shown in Fig. 5 over time period equivalent to 1/e of fluorescence decay, *i.e.*
3
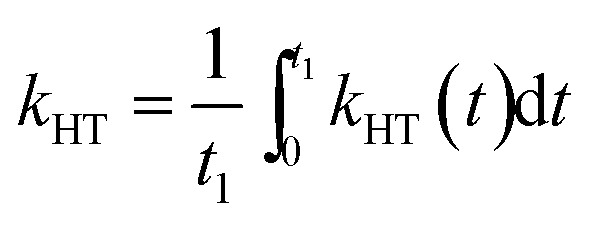
where *t*_1_ is the time for the fluorescence decay to fall to 1/e of its initial value. This results in the *k*_HT_ values in the range from 4 × 10^7^ to 8 × 10^8^ s^–1^, which means that the hole transfer in the studied inverted systems NiO/QD is fast and comparable to previously observed electron transfer rates (*k*_ET_ = 10^7^ s^–1^) in “classical” systems, such as TiO_2_/CuInS_2_.^42,51,53^ At the same time, as mentioned before the hole transfer to the electrolyte is much slower in classical systems with *k*_HT_ of 2–3 orders of magnitude lower compared to *k*_ET_.^10,54^ Very few data is available on hole transfer to NiO in the inverted sensitized systems with two recent works reporting on the hole transfer from CdSe to NiO with the rates in the range of 10^8^ to 10^9^ s^–1^,^29,30^ which are close to the ones observed in this work.

The observed hole transfer is about 15 times faster in the case of mixed selenide-sulfide QDs (Table 1). One of the possible reasons explaining this important difference is better energy alignment at the interface NiO/QD allowing for higher driving force for the hole transfer: introduction of selenium atoms raises the valence band of the CuInS_2_ QDs approaching it to the VB of NiO.

It is known that under the conditions of high excitation fluence and slow regeneration of the oxidized QDs upon electron transfer in n-type QDSSCs, formation of positive excitons (trions) is possible. Such trions decay subsequently by Auger recombination with a similar rate to that determined for electron transfer, which may lead to confusion.^55^ In the case of CuInS_*x*_Se_2–*x*_ QDs the decay lifetime of negative trions has been recently determined to be 230 ps resulting in an Auger recombination rate of 4.3 × 10^9^ s^–1^.^55^ This means that under the experimental conditions used here hole transfer appears on a longer timescale than negative exciton decay.

### Photovoltaic studies

**Fig. 6:**
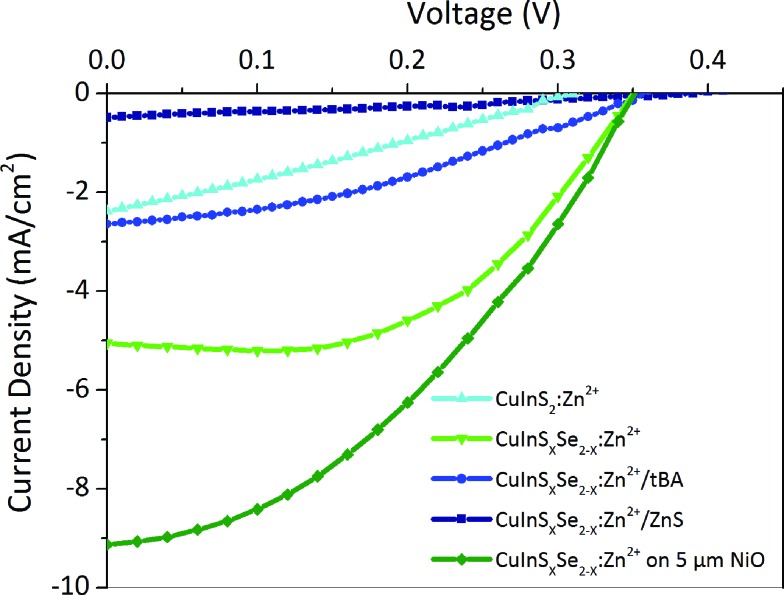
*J*–*V* curves of some solar cells tested.

Solar cells were fabricated from the best sensitized NiO substrates using aqueous polysulfide electrolyte used in conventional n-type QD sensitized cells. This electrolyte is capable of shuttling both electrons and holes in substitution of iodide electrolytes widely used in DSSCs, which lead to the photocorrosion of the QDs and severely limit the cell lifetime. Copper sulfide deposited on brass was used as a counter-electrode instead of platinum used in DSSCs as these are known to be poisoned by polysulfides. During the measurements, for simplicity the electrodes of the resulting cells were connected inversely compared to n-type QD sensitized cells. As a result, recorded *I*–*V* curves had a shape similar to classical cells developing positive current at positive polarization above open circuit voltage, positive *V*_oc_, and negative short circuit current, *J*_sc_ (Fig. 6). At least three samples were tested and their characteristics were averaged for each value in Table 2.

**Table 2:** Photovoltaic efficiencies of the cells tested under simulated solar light (AM1.5G conditions). The average characteristics of 3 cells are presented for each line unless stated otherwise

QDs	Surface treatment	NiO thickness [μm]	*J*_sc_ [mA cm^–2^]	*V*_oc_ [V]	FF	Efficiency [%]
CuInS_2_:Zn^2+^	—	3.5	1.54	0.33	0.28	**0.14**
CuInS_*x*_Se_2–*x*_:Cd^2+^	—	3.5	0.66	0.34	0.32	**0.07**
CuInS_*x*_Se_2–*x*_:Zn^2+^	—	3.5	5.72	0.34	0.41	**0.80**
			5.05	0.35	0.53	**0.95^*a*^**
CuInS_*x*_Se_2–*x*_:Zn^2+^	—	5.0	7.50	0.35	0.35	**0.91**
			9.13	0.35	0.39	**1.25^*a*^**
CuInS_*x*_Se_2–*x*_:Zn^2+^	MPA *ex situ* ^*b*^	3.5	0.87	0.46	0.25	**0.10**
CuInS_*x*_Se_2–*x*_:Zn^2+^	*t*BA *ex situ*	3.5	1.64	0.38	0.34	**0.21**
CuInS_*x*_Se_2–*x*_:Zn^2+^	NiO-MPA	3.5	0.96	0.45	0.25	**0.11**
CuInS_*x*_Se_2–*x*_:Zn^2+^	S^2–^ *in situ*	3.5	1.03	0.35	0.33	**0.12**
CuInS_*x*_Se_2–*x*_:Zn^2+^	ZnS	3.5	0.36	0.38	0.28	**0.04**

^*a*^Results for the best cell in series.

^*b*^ *Ex situ* stands for the ligand exchange in the solution of the QDs prior to deposition; *in situ* indicates ligand exchange on the QDs deposited on NiO and “NiO-MPA” indicates treatment of NiO by MPA prior to the deposition of QDs with pristine ligands.

In general, the cells demonstrate high short circuit currents of 2–9 mA cm^–2^, strongly dependent on the QD surface modifications, with relatively stable *V*_oc_ of 0.35–0.4 V and moderate fill factors of 0.35 (Table 2). The latter is related to charge recombination at interfaces and appears to be the inherent problem of all nanostructured semiconductor NiO films. They exhibit a high density of states in the band gap above the valence band, which act as traps for photogenerated carriers.^56,57^ The cells obtained with 3.5 μm thick NiO and CuInS_*x*_Se_2–*x*_:Zn^2+^ develop a high *J*_sc_ of 5 mA cm^–2^ together with a *V*_oc_ of 0.35 V and a fill factor of 0.53 resulting in an efficiency of 0.80% in average (with the best cell showing 0.95%) (Fig. 6). Cation exchange of the QDs with Zn^2+^ and Cd^2+^ plays an important role for the surface passivation and consequently conversion efficiency as non-exchanged QDs result in poor performance of the cells. By optimizing different parameters of the cells' components we attempted to improve this performance. Not surprisingly, we were not able to modify substantially the open circuit voltage, which is generally defined by the energy difference of the valence band of the photocathode (NiO) and the redox level of the electrolyte (polysulfide). At the same time, the effect of different treatments of the QDs on the obtained photocurrents is significant. Generally, the cells based on QDs containing selenium result in higher *J*_sc_ and constant *V*_oc_ and fill factor, and thus higher photovoltaic efficiencies compared to pure CuInS_2_ QDs. This is in agreement with photophysical studies showing much slower hole injection of the latter compared to CuInS_*x*_Se_2–*x*_. Better-aligned energy levels in the case of mixed sulfide-selenide QDs could be at the origin of this behavior. Other possible reasons contributing to the better photovoltaic performance is the larger absorption range of mixed sulfide-selenide QDs. Zn^2+^ cation exchanged QDs equally result in higher obtained photocurrent compared to Cd^2+^ exchanged ones due to better surface passivation and lower charge recombination.

The homogeneous coating of the pores of a 3.5 μm thick NiO mesoporous layer by QDs seen on STEM EDX images (see Fig. 3) allows assuming that QD penetration is not limited by the thickness of NiO. In order to increase the QD loading on the NiO scaffold, we have decided to increase its thickness. For cells with a 5 μm thick NiO mesoporous layer the efficiency further augmented reaching the value of 1.25% for the champion cell, which is to the best of our knowledge the highest efficiency for any inorganically sensitized p-type solar cells. This comparably high efficiency is primarily due to the unprecedented *J*_sc_ increased from 5.05 (for thinner cells) up to 9.13 mA cm^–2^. The higher *J*_sc_ observed for thicker electrodes allows also understanding the influence of light penetration into the cells: it is known that one of the major drawbacks of mesoporous NiO electrodes is their opacity because of various impurities, such as Ni^0^.^48^ This opacity can lead to a decreased light-harvesting efficiency (LHE) and the capacity of light absorption by the sensitizers. The fact that the short circuit current significantly grows upon the increase in photocathode thickness reveals that the light generated by AM1.5G solar simulator penetrates at least up to 5 μm into the optimized NiO scaffold confirming that the cell performance is not limited by its thickness. It is worth mentioning that NiO electrodes with thickness above 5 μm are increasingly hard to fabricate because of the mechanical constraints.

Surface ligands play an important role in the charge transfer both to the wide gap electrode and to the redox electrolyte.^58,59^ Long organic chains passivating the QD surface after the synthesis prevent an efficient transfer in QD sensitized solar cells because they create a physical and energy barrier for the charge carriers. A typical strategy is thus to replace them with shorter ligands using either an *ex* or *in situ* approach.^8^ Ligand exchange strategies were applied to replace original long dodecanethiol and oleylamine ligands passivating the surface of the QDs used in this work. *Ex situ* stands for the ligand exchange in the solution of the QDs prior to deposition; *in situ* indicates ligand exchange on the QDs deposited on NiO and “NiO-MPA” indicates treatment of NiO by mercaptopropionic acid (MPA) prior to the deposition of QDs with pristine ligands. Contrary to conventional n-type cells, where they were shown to work efficiently,^6,60^ short capping ligands (MPA; *tert*-butylamine, *t*BA; sulfide anions) when applied to inverted systems lead to a decreased photovoltaic performance, especially due to a significantly worse *J*_sc_ after the exchange although the *V*_oc_ can be sometimes higher (in the case of MPA treatment). Several reasons can be proposed to explain this decrease of photovoltaic performance as a consequence of surface modifications: less efficient surface passivation, perturbation of energy levels, and change in QD loading. Less efficient QD surface passivation by the new ligands can indeed result in higher losses related to unpassivated surface traps. In the case of “classical” QDSSCs this effect is largely compensated by a gain in charge (hole) transfer to the electrolyte, however probably it is not the case for the electron transfer for the inverted cells. A shift of QD energy levels as a function of passivating ligands can be an additional reason for the efficiency loss. It is known that in some cases ligand exchange can lead to a shift of valence and/or conduction bands of the QDs because of the strong coupling between the ligand and QD surface atoms with consequent electronic perturbation.^61,62^ As a result, electronic levels modification upon ligand exchange might lead to less favorable energy alignment for the charge transfer. In order to check this hypothesis and estimate the energy levels of colloidal QDs before and after the ligand exchange, electrochemical studies have been performed. Differential pulsed voltammetry (DPV) reveals that the shift of energy levels as a result of surface modification is not very pronounced (less than 0.1 eV) (Table 3), therefore, it is not expected that the ligand exchange would have a significant effect on the band alignment and charge injection at the interfaces with NiO and electrolyte. At the same time, slightly higher increase of the conduction band energy of *t*BA-coated QDs can cause less efficient charge transfer to the electrolyte compared to native dodecanethiol (DDT)-coated QDs as a result of the increased energy difference with the polysulfide couple redox potential (4.1 eV).

**Table 3:** Electronic energy levels of CuInS_*x*_Se_2–*x*_:Zn^2+^ QDs determined by DPV studies

QD/ligands	Native (DDT)	MPA	*t*BA
*E*_VB_, eV	5.43	5.44	5.46
*E*_CB_, eV	3.54	3.51	3.42

It is worth noting that electronic level alignment at the interface with NiO and electrolyte in real devices may be different because of band bending and levels pinning. Additional studies to probe such interfaces by UPS are currently underway to give a more detailed answer based on which alternative strategies of ligand exchange could be developed. In addition, the method of QD surface treatment can also play a role in the performance in the cells: QDs processed using *ex situ* exchange possess the new ligands on the entire surface, while for the *in situ* exchanged ones only the surface not in contact with the substrate is modified.^8^ The studied systems, however, do not seem to depend on the order of ligand exchange: the negative factors described above probably dominate the overall performance.

Finally, the role of QD loading in the devices as a function of the surface treatment used was determined. From the EDX studies it was possible to find the ratio of the mass of CuInS_*x*_Se_2–*x*_ QDs to the overall mass of sensitized mesoporous NiO scaffold. The highest loading (15–16 mass%) was achieved using unmodified QDs with native DDT and oleylamine ligands, and *t*BA. Contrary to the case of CdSe sensitization of NiO,^31^ MPA pre-treatment of NiO and *ex situ* MPA ligand exchange was found to lead to much lower QD loading in the film (3–4 mass%) (see details in ESI†), which corresponds very well to much lower photocurrent measured in the corresponding cells. Lower loading achieved using NiO functionalization by MPA can be due to the lower penetration of the QDs to the pores of mesoporous scaffold, while *ex situ* MPA ligand exchange probably lowers the attainable concentration of the QD solution because of the decreased solubility of MPA coated QDs. Taken together, surface modification of QDs strongly influences the photovoltaic performance of the inverted QDSSCs essentially because of the changed QD loading with some potential contribution of the energy band modification.

**Fig. 7:**
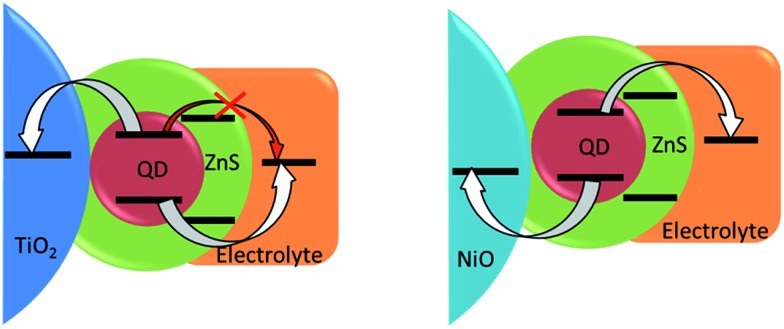
Scheme of energy levels alignment after inorganic ZnS passivation of QDs on standard (left) and inverted (right) configurations.

Another approach developed to optimize the charge transfer for the conventional QDSSCs and thus improve their photovoltaic performance consists in inorganic coating of the sensitized electrodes by SILAR. While the surface of QDs already contains a passivation layer due to the presence of Zn^2+^ cations, it probably represents only a thin (sub)monolayer and a thicker coating could be necessary for more efficient passivation. The ZnS layer deposited by SILAR is known to decrease the rate of recombination in n-type QDSSCs.^1,63^ The same strategy adapted in the case of p-type cells studied in this work turned out to be counterproductive: even though the *V*_oc_ slightly increased from 0.34 to 0.38 V, the almost 4-fold drop of the *J*_sc_ has wiped out any positive effects of the ZnS coating leading to a more than triple decrease in the cell efficiency. The major role of the wide band gap ZnS coating in n-QDSSCs is to suppress undesired charge recombination between the electron on the conduction band of excited QD and the redox level of the polysulfide electrolyte (Fig. 7). However, in the case of p-type QDSSCs, electron transfer from the QD to the electrolyte is an integral part of the working principle of the photovoltaic process. Therefore, by introducing a high-lying conduction band of ZnS we render the electron transfer less efficient, which is manifested by a decreased photovoltaic efficiency. While a thin layer of Zn^2+^ has a positive effect, a SILAR-grown layer is probably too thick to allow for tunneling. Similar dependence of the solar cell efficiency on the thickness of the passivation layer on CuInS_2_ QDs (in the case of n-type cells) has been observed by other groups.^6,64,65^ By consequence, other strategies are needed to suppress undesired recombination pathways originating here from hole transfer from the QD valence band to the electrolyte. A more n-type material showing type II band alignment with respect to the CuInS_2_Se_2–*x*_ could be an option.

IPCE spectra of the cells generally followed the shape of the absorption spectra of CuInS_2_Se_2–*x*_ QDs confirming that the photogenerated current is indeed due to the QDs (Fig. S6 and S12†). The cells demonstrate efficient charge generation (up to 72% efficiency at 390 nm) over the broad absorption range up to 900 nm. By integrating the IPCE spectra over the full wavelength range short-circuit photocurrents can be estimated. For the CuInS_2_Se_2–*x*_ sensitized NiO cell integrated IPCE values yield the current of 5.30 mA cm^–2^, which is lower than the real *J*_sc_ value measured in the cell (7.50 mA cm^–2^). Similar phenomena between the estimated and measured photocurrents have been already observed in the case of both n-^66^ and p-type QDSSCs.^33^ To understand the origin of this discrepancy the techniques of measurement under IPCE and simulated sunlight conditions need to be compared. The IPCE for the sensitized cells is defined^67^ by the product of three parameters: LHE of the sensitizer (QDs), charge injection yield (hole injection from the excited QDs to the NiO) and charge collection efficiency by the back contact. The LHE of a QD sensitized cell according to Lambert–Beer law is determined by the QD loading, their extinction coefficient, and the optical absorption depth in the NiO film. While most of the parameters listed above basically stay constant for the two techniques, the absorption depth can vary considerably as a function of the light source used. Indeed, as mentioned before, the mesoporous NiO electrode is much less transparent compared to TiO_2_, therefore the intensity of incident light could play an important role on its penetration depth and the intensity of the simulated sunlight (100 mW cm^–2^) is a thousand times stronger compared to the monochromatic light used for IPCE measurements (env. 100 μW cm^–2^). After the noise correction, the real IPCE spectrum for an inverted QDSSC exhibits the expected shape (Fig. S12†). Moreover, upon the integration the spectrum yields a *J*_sc_ of 7.22 mA cm^–2^, which is very close the current measured for the cell (7.50 mA cm^–2^), which confirms our hypothesis about the origin of the initial short circuit current discrepancy.

## Conclusions

In conclusion, we have presented a detailed study of p-type QDSSCs sensitized with Cd- and Pb-free CuInS_2_Se_2–*x*_ nanocrystals. By using an optimized formulation and deposition of the mesoporous NiO electrode and CuInS_*x*_Se_2–*x*_ QDs with tailored electronic properties, efficient sensitized electrodes were obtained. The QDs show an excellent penetration and loading on the NiO electrode. Photophysical studies of the assemblies demonstrate very fast hole injection from QDs into NiO reaching 8 × 10^8^ s^–1^, which is comparable to the electron injection rate in classical n-QDSSCs. By optimizing the materials and fabrication conditions a photovoltaic efficiency of 1.25% was obtained, which is the highest value reported so far for p-type QDSSCs. The ligand exchange strategies and blocking layer deposition *via* SILAR widely used for the improvement of conventional n-type QDSSCs turn out to be inefficient in the case of inverted architectures because of different operating mechanisms involved. The results presented here pave the way for the design of new efficient p-type solar cells sensitized by non-toxic and low cost QDs, with a potential to outperform the p-type dye-sensitized cells (*η* = 2.5%) and to be used in tandem cells with optimized n-type subcells.

## Experimental part

### Materials

Indium acetate (In(OAc)_3_, 99.99%), copper iodide (CuI, 99.99%), dodecanethiol (DDT, 98%), 3-mercaptopropionic acid (MPA, 99%), *tert*-butylamine (*t*BA, 99.5%), octadecene (ODE, 90%), cadmium oxide (CdO, 99.5%), zinc oxide (ZnO, 99+%), sodium sulfide nonahydrate (Na_2_S·9H_2_O, 98%) were purchased from Sigma-Aldrich. Oleylamine (OAm, 80–90%) and zinc nitrate hexahydrate (Zn(NO_3_)_2_·6H_2_O, 98%) were from Acros. Sodium sulfide anhydride (Na_2_S), trioctylphosphine (TOP, 90%) and oleic acid (OA, reagent grade) were obtained from Alfa-Aesar, Fluka and Fisher, respectively. All chemicals were used as received without further purification.

#### Synthesis of CuInS_*x*_Se_2–*x*_ QDs

1 mmol of indium acetate is mixed with 1 mmol of copper(i) iodide, 5 ml of 1-dodecanthiol and 1 ml of oleylamine in a three-neck flask. The reaction mixture is degassed under vacuum for 10 min and purged with argon three times, then the flask is heated to 100 °C until the solution became yellow and transparent. The flask is then slowly heated to a growth temperature up to 230 °C, and at a temperature of 220 °C, slow injection of 6 mmol of 2 M TOP-Se begins. After 30 min the reaction mixture was cooled and the QDs were purified by addition of 5 ml of chloroform and precipitation with 10 ml of methanol. The precipitated QDs were collected by centrifugation at 6000 rpm for 5 min and the supernatant was discarded. The QDs were stored in 5 ml of chloroform following purification.

#### Cation exchange and recapping of QDs^6^

For cation exchange with Cd^2+^ or Zn^2+^, a stock solution of 0.5 M cadmium or zinc oleate was prepared with 3 : 1 = oleic acid : CdO or ZnO dissolved in ODE. 5 ml of the purified QDs in chloroform solution were added to 5 ml of 0.5 M Cd/Zn-oleate solution and the reaction mixture was refluxed for 10 min. Following cation exchange, the chloroform was added to the reaction solution, and then 10 ml of acetone was added to precipitate the QDs. The precipitated QDs were collected by centrifugation at 6000 rpm for 5 min, and then dissolved in 5 ml of chloroform.

#### QDs surface treatment

The ligand exchange was prepared following slightly modified methods of H. McDaniel *et al.* ^6^ and J. Aldana *et al.* ^68^

#### *Ex situ* exchange

For *t*BA recapping, the ligand was used as a solvent to dissolve the precipitated QDs. Then, methanol was added to precipitate the QDs, which were collected by centrifugation and the supernatant was discarded. The ligand was again used as a solvent to dissolve the precipitated QDs and these solutions were sonicated for a few minutes at room temperature. Methanol was added to precipitate the QDs, which were collected by centrifugation. The above process was optimized to give the highest QD-loading in the mp-TiO_2_ film for *t*BA-capped QDs. The recapped QDs were dissolved in chloroform and the solution was centrifuged to remove large aggregates that occasionally formed during recapping. Any precipitate was discarded. For MPA recapping, the QDs were recapped by dissolving precipitated QDs in a mixture of 1 : 1 chloroform and MPA, then precipitating by adding methanol, and centrifuged. The supernatant was discarded, and the QDs were dissolved in the same total volume of MPA and methanol (1 : 1). The solution was sonicated for a few minutes. Chloroform was added to precipitate the MPA-capped QDs, and the QDs were collected by centrifugation. The MPA-capped QDs were dissolved in methanol, and the solution was centrifuged to remove aggregates that formed during recapping and to remove partially recapped QDs.

#### NiO MPA treatment

NiO electrodes were immersed for 12 h into a solution containing MPA (1 M) and sulfuric acid (0.1 M) in acetonitrile^69^ followed by thorough rinsing with methanol. The MPA-modified NiO electrodes were then immersed in the QDs solution for 24 h to ensure saturated entrapment of the QDs onto the functionalized NiO electrodes.

#### *In situ* exchange

For S^2–^ recapping^60^ the sensitized electrode was dipped into Na_2_S solution in formamide (10 mg mL^–1^) for 1 h in the dark under inert atmosphere. After exchanging, the electrodes are rinsed with methanol.

#### ZnS SILAR

0.1 M of Zn(NO_3_)_2_ solution in ethanol and 0.1 M of Na_2_S solution were used as cationic and anionic sources, respectively. Each cycle consisted of 1 min per dipping and washing with proper pure solvent between each step. In total, two cycles were performed for the cells.

### Device fabrication

The photocathodes were prepared by doctor blading NiO film of various thickness (3.5 ± 0.4 μm and 5 μm ± 0.5 μm) with Ni-nanoxide paste (Solaronix) on FTO substrates and sintering at 400 °C for 30 min. The films were immersed in each QDs solution for 4 days. The films were then rinsed with pure chloroform to remove unattached QDs. The cells were constructed by assembling the brass-based Cu_2_S counter electrode and QD-sensitized NiO films electrode using Parafilm (PM-996) as a spacer with a binder clip. The Cu_2_S counter electrodes were prepared by first activating the brass foil in 1.0 M HCl solution at 85 °C for 30 min followed by the reaction with the electrolyte. Polysulfide aqueous solution is used as electrolyte, consisting of 1.0 M Na_2_S, 1.0 M S, and 0.1 M NaOH. The device active area was 0.6 × 0.6 cm (that is, 0.36 cm^2^).

### Characterization

Current–voltage characteristics and power conversion efficiencies of the solar cells were measured under inert atmosphere using a computer controlled Keithley 2400 unit and 1000 W m^–2^ air-mass 1.5G simulated solar light generated by a Newport class AAA solar simulator. A calibrated monocrystalline silicon solar cell (P/N 91150V from Oriel) was used as a reference. For incident-photon-to-electron conversion efficiency (IPCE) the samples were illuminated with a mercury-xenon lamp through a Newport 74125 Cornerstone 260 monochromator. Photocurrent measurements were performed with a Newport 70104 Merlin digital lock-in radiometry system. XPS spectra were acquired using M-XPS system from Omicron Nanotechnology (Taunusstein, Germany) with a seven-channel hemispheric electron analyzer (model EA125 U7 HR) providing high energy resolution (210 meV). TEM images were acquired by a FEI Tecnai Osiris (S)TEM microscope operated at 200 kV. Scanning transmission electron microscopy (S-TEM) observations have been done using an FEI Osiris microscope operated at 200 kV or 80 kV. This microscope is fitted with a high brightness gun and with four windowless silicon drift detectors for the X-ray spectroscopy, resulting in a large collection angle of about 0.9 sr, which allows efficient acquisition of EDX spectral images. Quantitative elemental maps are then calculated using the Cliff–Lorimer ratio technique. For STEM observations, the NiO layers are prepared in thin lamella by micromachining with a focused ion beam microscope, with a final thinning step at 2 kV and a targeted thickness of 100–150 nm.

Focused ion beam (FIB) tomography has been realized in a Zeiss NVision 40 dual-beam instrument. In this technique, the NiO layer is cut in cross-section, slice by slice, with a Ga^+^ ion beam (with a 700 nA current at 30 kV), and each slice is imaged in scanning electron microscopy (SEM) at 5 kV using the in-chamber secondary electron detector. To minimize curtaining effects, a 1 μm thick carbon layer is used prior to the etching in order to smooth the surface of the sample. 400 slices of 4k × 4k pixels have been acquired, with a slice thickness of 3 nm and a pixel size of 3 nm for SEM images.

Electrochemical DPV measurements were performed inside a glove box using an Autolab3 potentiostat/galvanostat using Ag wire pseudo-reference electrode and Pt counter electrode and working electrode (diameter: 4 mm). The samples were prepared by drop-casting 10 μL of a 20 mg mL^–1^ QD colloidal solution in chloroform on the working electrode and subsequent immersion of the electrodes in the solution of electrolyte (0.1 M tetrabutylammonium hexafluoride in acetonitrile).

#### Photophysical studies

Thinner transparent NiO substrates (900 nm) were used for the photophysical studies. UV-vis spectroscopy was performed with a Hewlett Packard 8452A and Varian Cary 300 spectrophotometers. Steady-state emission spectra were measured by means of a Hitachi F-4500 and Edinburgh Photonics FLS980 instruments. For PL measurements, an excitation wavelength of 375 nm was used. Time-resolved photoluminescence spectroscopy was carried out at an excitation wavelength of 375 nm using a PicoQuant picosecond pulsed laser and PL was detected using time correlated single photon counting (TCSPC) with time resolution of ∼200 ps.
