# Supplementary material for: Efficient eco-friendly inverted quantum dot sensitized solar cells
Source: J Mater Chem A Mater. 2015 Dec 1;4(3):827–37. doi: 10.1039/c5ta06769c (PMC4936380; doi:10.1039/c5ta06769c)
Supplement: Supplementary file 1 [file TA-004-C5TA06769C-s001.pdf]

Electronic Supplementary Material (ESI) for Journal of Materials Chemistry A.
This journal is © The Royal Society of Chemistry 2015

Electronic Supplementary Information

Efficient eco-friendly inverted quantum dot sensitized solar cells

Jinhyung Park,[a,b,c] Muhammad T. Sajjad,[d] Pierre-Henri Jouneau,[e] Arvydas Ruseckas,[d] Jérôme Faure-Vincent,[a,b,c] Ifor D. W. Samuel,*[d] Peter Reiss [a,b,c] and Dmitry Aldakov* [a,b,c]

[a] Univ. Grenoble Alpes, INAC-SPRAM, F-38000 Grenoble, France, dmitry.aldakov@cea.fr

[b] CNRS, INAC-SPRAM, F-38000 Grenoble, France

[c] CEA, INAC-SPRAM, F-38000 Grenoble, France

[d] Organic Semiconductor Centre, SUPA, School of Physics and Astronomy, University of St Andrews, North Haugh, St Andrews, Fife, UK, idws@st-andrews.ac.uk

[e] CEA, INAC-SP2M, LEMMA, F-38000 Grenoble, France

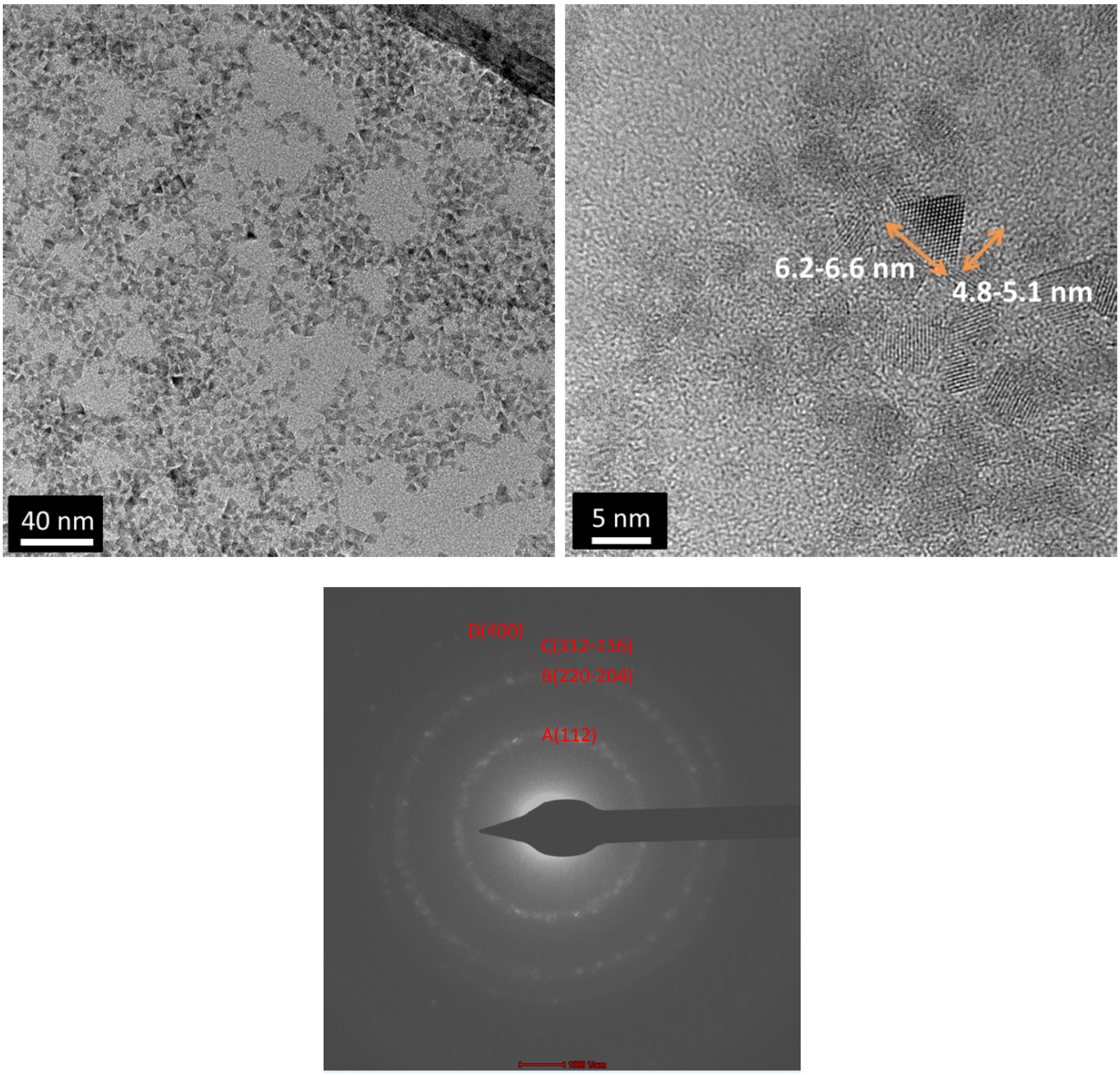

CL 1m

Lattice parameter (Angström)	$CuInS_2$	$CuInSe_2$	SAED measurements
**a**	5.52	5.78	6.3 ± 0.4
**c**	11.12	11.55	12.4 ± 0.8

Figure S1. TEM images of $CuInS_XSe_{2-x}$ QDs (top panels); SAED pattern of the same QDs (middle panel) with the extracted lattice parameters (lower table).

Porosity estimation by SEM image segmentation. The image stack of 400 slices of 4k*4k pixels with a slice thickness of 3 nm and a pixel size of 3 nm for SEM images has been registered by cross-correlation using the StackReg plugin in the Fiji software. This gives us directly an image the 3D structure of the sample with an isometric voxel size of 3x3x3 nm. The layer appears has highly porous isotropic foam with cells of about 100 nm with very thin (< 10 nm) walls. Basic threshold

segmentation, with a subsequent filtering to remove individual voxel, was then realized with the FEI Avizo software in order to extract the material volume, which allows estimating the porosity. At the same time, it has to be noticed that volume segmentation presents some artefacts due to high pore volume of the material that may lead to a slight underestimation the porosity. This is due mainly to the so-called "shine through" artefact in the FIB-SEM image stack, resulting from the fact that the bottom of the pores is visible below the cutting plane in SEM images. An advanced (but difficult to implement) segmentation procedure has recently been proposed for this type of images, and is expected to permit a higher accuracy in the quantitative analysis.[S1]

Alternative estimation of the porosity from the weight of the porous NiO has been made the following way: a substrate of mesoporous NiO with a known surface was scratched and its mass was measured. Knowing the thickness of the NiO layer, it was possible to estimate the total volume of mesoporous NiO occupied by a gram of film. From the density of bulk NiO a volume occupied by solid NiO per gram of film was determined. The difference between the total volume of mesoporous material and volume of solid NiO represents the pore volulme.

Table S1. Composition of the QDs studied determined by EDX

	Cu	In	S	Se	Zn	Cd
**$CuInS_xSe_{2-x}$**	17.4	34.8	8.5	39.1	-	-
**$CuInS_xSe_{2-x}:Zn^{2+}$**	15.1	33.4	8.3	33.0	10.3	-
**$CuInS_xSe_{2-x}:Cd^{2+}$**	16.9	27.9	12.7	21.5	-	21.0

QD loading was determined from the EDX composition of the films ignoring the organic contribution (ligands, linkers and impurities) by taking the ratio between the mass percent of QDs and the sum of contributions of masses of NiO and QD.

Table S2. QD loading in the sensitized NiO films as a function of surface treatments

	**$CuInS_xSe_{2-x}:Zn^{2+}$**				**$CuInS_xSe_{2-x}:Cd^{2+}$**
	**DDT**	**MPA**	**tBA**	**NiO-MPA linker**	**DDT**
**Loading, mass %**	16	4	15	3	4

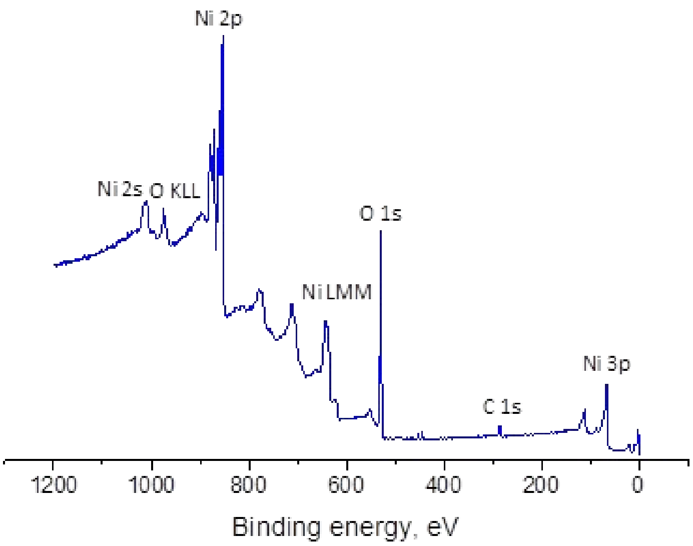

Figure S2. XPS survey spectrum of mesoporous electrode before the deposition of the QDs: only elements related to the NiO are present.

Interpretation of XPS spectra of transition metals is generally considered as challenging due to the complex multifeature spectra including shake-up and plasmon loss peaks overlapped with contributions of peaks of different oxidation. The high resolution XPS spectrum of the Ni 2p region of mesoporous NiO can be deconvoluted using 5 peaks according to the fitting model proposed by Biesinger et al. established for pure NiO powder (Table S1).[S2] Positions and relative contributions of each peak are in excellent agreement with the reference material studied in the described paper. Typical position of the peak of metallic Ni (852.6 eV) is given by a dashed line for comparison.

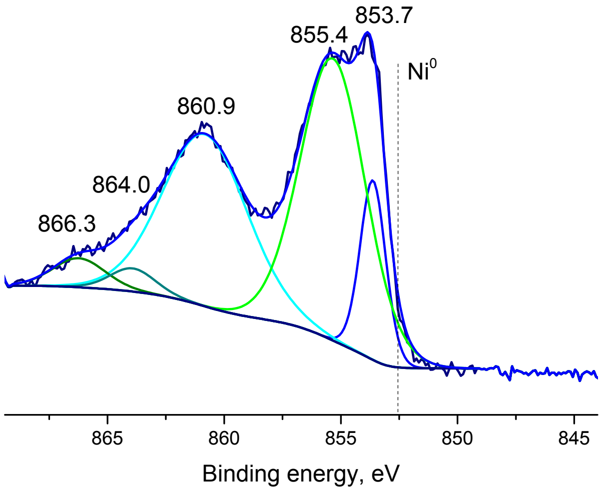

Figure S3. High resolution XPS spectrum of the Ni 2p region of NiO.

Table S3. Deconvolution parameters of different Ni 2p peaks

NiO peaks	Peak 1	Peak 2	Peak 3	Peak 4	Peak 5
Peak position, eV	853.7	855.4	860.9	864.0	866.3
%	11.4	45.7	36.5	2.6	3.8

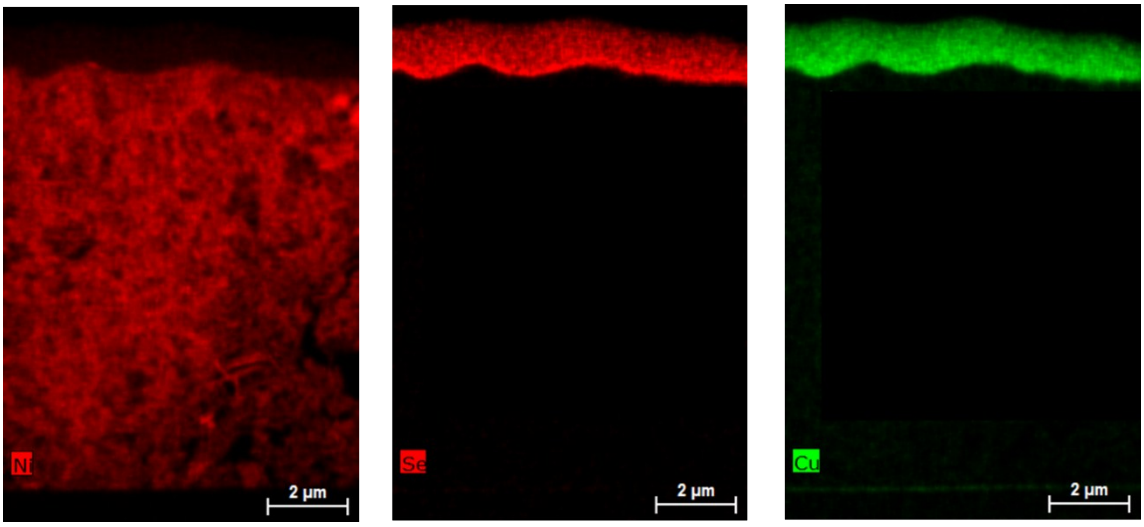

Figure S4. STEM EDX cross-section images of a mesoporous NiO layer showing absence of Se and Cu elements in the electrode before the QD deposition. The apparent "glowing" of the NiO layer is considered as an artefact related to the measurement technique.

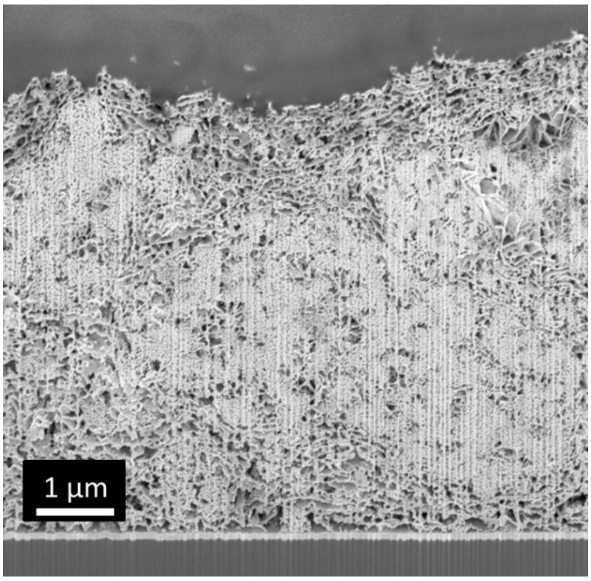

Figure S5. SEM cross-section image of mesoporous NiO layer with deposited $CuInS_XSe_{2-x}$ QDs

Photophysical studies: The absorption spectra of $CuInS_2$:Zn^{+2} and $CuInS_xSe_{2-x}$: Zn^{+2} on glass and NiO are shown in Fig. S6. For comparison, absorption spectrum of NiO is also plotted. Both QDs show broad absorption in the range of 300-700 nm which is typical features of these systems. However, the $CuInS_xSe_{2-x}$: Zn^{+2} show higher absorption in the rage of 300-500 with a shoulder around 400 nm. There are some extra features appeared when QDs are deposited on to NiO (Fig. S6, right). Photoluminescence (PL) spectra of $CuInS_2$:Zn^{+2} on glass and NiO (given in Fig. S6) show a weak broad emission (around 850 nm) instead of band edge emission, which corresponds to donor-acceptor pair recombination.[S3,S4]

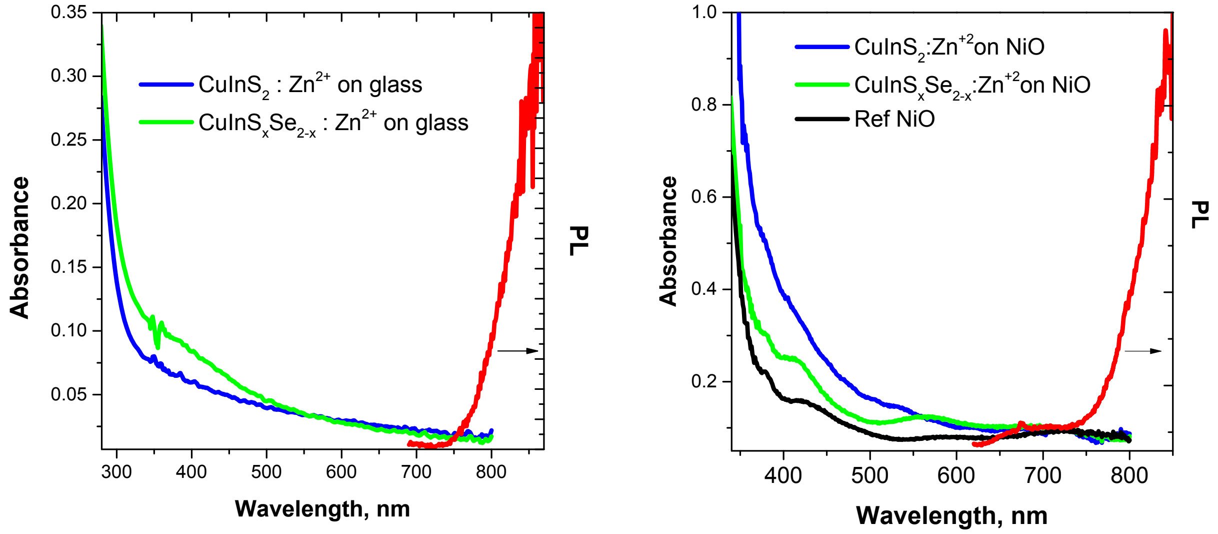

Figure S6. Absorption and emission spectra of QD films on glass (left) and on NiO (right). PL emission was obtained by exciting sample at 400 nm.

In order to investigate the charge transfer process from QDs to NiO, we have performed time-resolved photoluminescence spectroscopic studies of QDs deposited on glass and NiO substrates. First, PL decays of $CuInS_2$:Cd^{2+} and $CuInS_2$:Zn^{2+} QDs on glass and NiO substrates were measured and resulting graphs are shown in Figure S7. The PL decays were multiexponential and were fitted with triexponential decay. Each decay component corresponds to different processes. The detail is given in the main manuscript. The average lifetime was calculated using weighted average and is given in Table S2 along with other fitting parameters.

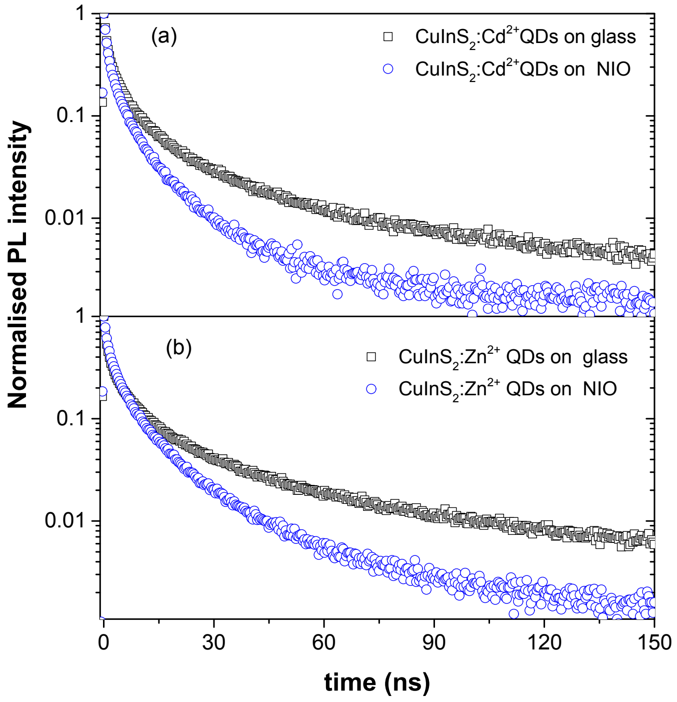

Figure S7. PL decays of $CuInS_2$ QDs on glass (black dots) and on NiO (blue dots). (a) PL decays of $CuInS_2:Cd^{2+}$ on glass and NiO; (b) PL decays of $CuInS_2:Zn^{2+}$

In the case of QDs on NiO substrate, a significant shortening of PL lifetimes was observed compared to those of QD films on glass (shown in Figure S7). This apparent reduction of lifetime is due to charge transfer (hole transfer) from excited QDs to NiO. We extracted this charge transfer rate from additional rate of decay of PL due to charge acceptor NiO which we assign to hole transfer from QDs to NiO. For that we take the natural logarithms of the ratio of PL decay on NiO to the PL decay on glass. The resulting graphs are shown in Figure S8 whose slopes are equal to hole transfer rate[S5]

$$k_{HT}(t) = -\frac{d}{dt}\left(ln\left(\frac{PL\ of\ QD\ on\ NiO}{PL\ of\ QD\ on\ glass}\right)\right)$$

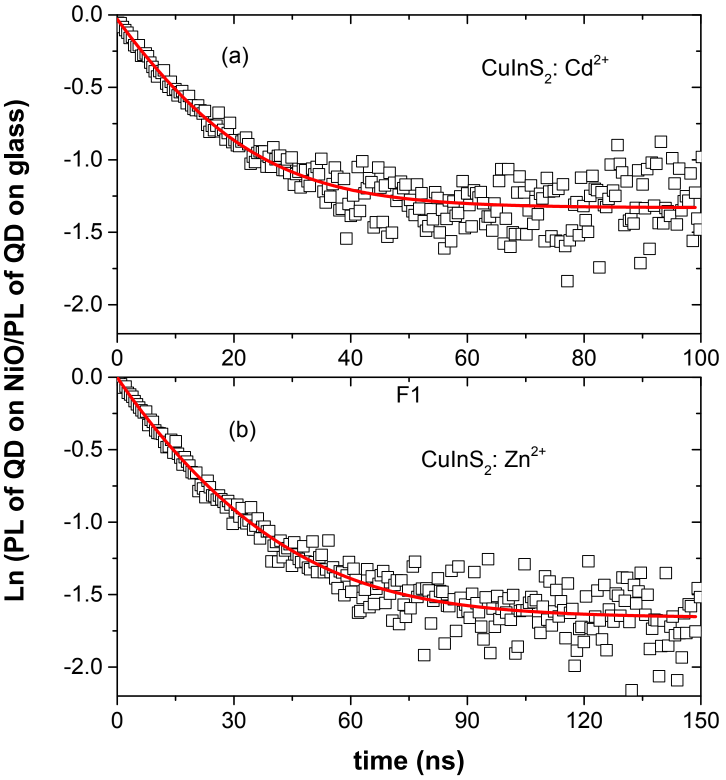

Figure S8. Natural logarithm of ratio of PL decay of $CuInS_2$ on NiO to PL decay on glass. The red lines are simulated curve used to calculate the hole transfer rate (detail is given in the text).

However, the data shown in Figure S8 was very noisy. Therefore, we fitted it before differentiation (red lines in Figure S8). Then we differentiated these simulated curves (red curves) to determine the hole transfer rates. The resulting hole transfer rates are shown in Figure 5(b) of main manuscript. We found that hole transfer rates are strongly time-dependent and cannot be described by single value. Therefore, for comparison with other studies, we determine the average hole transfer rates by integrating time-dependent hole transfer rate over time period of 1/e of fluorescence decay. The detail is given in the main manuscript. Our approach of finding the hole transfer rate is unique and different from previous studies which assume monoexponential decay. Similarly, the PL decays of $CuInS_xSe_{2-x}$:Cd^{2+} on glass and NiO was measured. Similar data analysis (described above) was used to extract the time- dependent and average hole transfer rate.

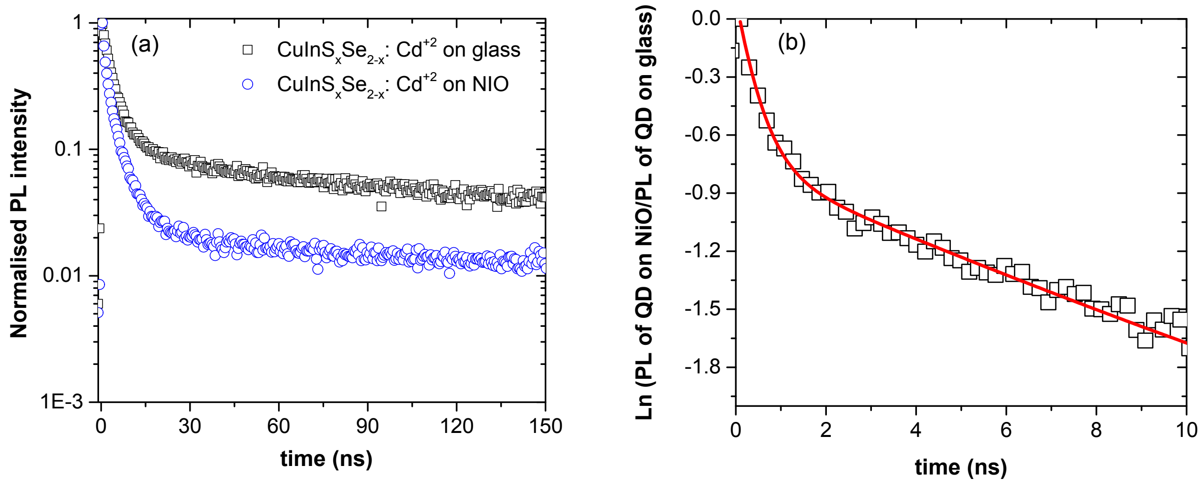

Figure S9 : (a) PL decays of $CuInS_xSe_{2-x}$:Cd^{2+} QDs on glass (black dots) and on NiO (blue dots). (b) Natural logarithm of ratio of PL decay of $CuInS_xSe_{2-x}$: Cd^{2+} on NiO to PL decay on glass. The red line is a fitted curve used to calculate the hole transfer rate.

Table S4. Triexponential fit parameters for the PL decays of QDs deposited on various substrates

QDs	Substrate	α_1	τ_1, ns	α_2	τ_2, ns	α_3	τ_3, ns	τ_{av}, ns
**$CuInS_2$:Cd^{2+}**	Glass	0.20	1.0	0.44	9	0.36	69	29
	NiO	0.20	0.4	0.46	3	0.34	15	7
**$CuInS_2$:Zn^{2+}**	Glass	0.20	1.7	0.39	17	0.41	168	76
	NiO	0.20	0.6	0.50	5	0.3	25	10
**$CuInS_xSe_{2-x}$:Zn^{2+}**	Glass	0.13	1.0	0.37	4	0.50	198	101
	NiO	0.28	0.4	0.55	4	0.17	34	8
**$CuInS_2Se_{2-x}$:Cd^{2+}**	Glass	0.12	1.8	0.16	5	0.72	100	73
	NiO	0.37	0.6	0.48	4	0.15	66	12

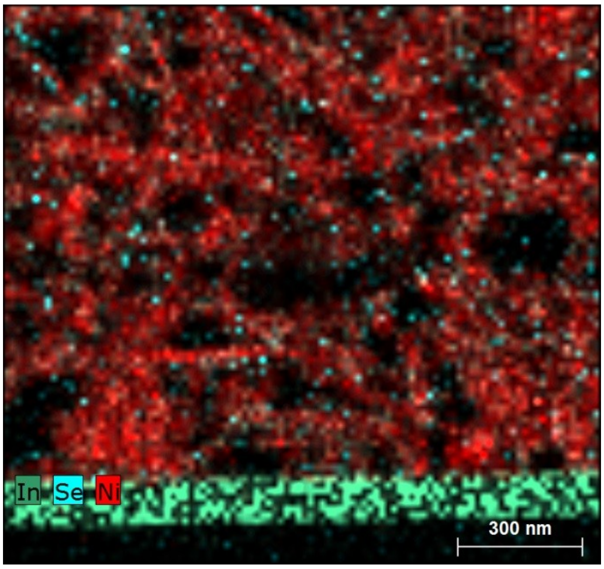

Figure S10. High resolution STEM EDX image of a mesoporous NiO layer showing the presence of Se and In elements in the vicinity of ITO electrode because of the penetrated QDs.

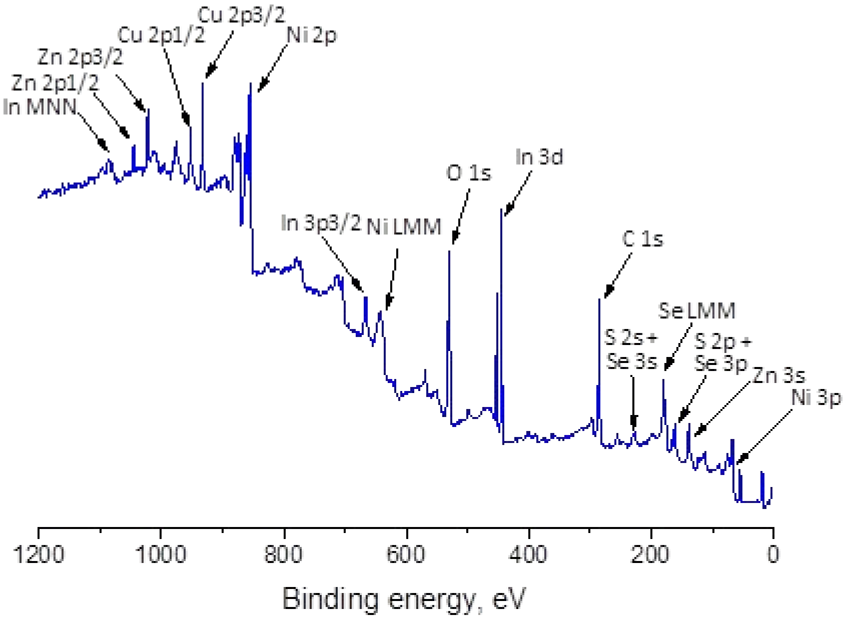

Figure S11. Survey XPS spectrum of mesoporous NiO with deposited $CuInS_xSe_{2-x}:Zn^{2+}$ QDs.

Non-exchanged quantum dots result in poor performance of the solar cells mainly because of the low short circuit current and fill factor probably because of the higher recombination losses, for example a cell based on $CuInS_xSe_{2-x}$ yields J_{sc} of 0.54 mA/cm^2, V_{oc} of 0.43 V and FF of 0.20 with the overall photovoltaic efficiency of 0.05%.

IPCE. The IPCE spectra recorded for the inverted QD sensitized cells used in this work have systematically an important contribution of noise manifesting as « drop » features, which are

especially present in the UV-visible region (Fig. S12). Similar features were observed before.[S65] Regardless of the cell type tested the features are positioned at specific wavelengths (360, 400, 440, 550, and 580 nm) leading to an assumption that this spectral character is related to the measurement technique rather than to a particular phenomenon of the cells. Indeed, the drops in the measure IPCE spectrum correspond almost exactly to the peaks of the excitation spectrum of the mercury-xenon lamp used for the IPCE measurement. Usually, these peaks are taken into account by measuring the reference spectrum using a Si photodiode resulting in smooth IPCE spectra, such as the one measured for a solar cell sensitized with an organic dye shown in the Figure S12 as a blue curve. However, as mentioned in the main text of the manuscript in the case of inverted QDSSCs, the excitation beam does not pass entirely through the cell because of the opacity of NiO/QD electrode and the efficient the absorption is therefore limited by a part of the cell. We consider this a major reason of the described unstable spectra character: when accounting for the excitation beam the software considers full transparency of the cell and applies its correction factors accordingly, which is not 100% compatible with the non-transparent devices studied in this work. We believe nevertheless that this overcorrection of the excitation peaks does not influence significantly the overall intensity and shape of the IPCE spectra. After the necessary noise correction for the excitation source, the real IPCE spectrum for an inverted QDSSC takes the expected shape (see red curve on Fig. S12). Upon integration the spectrum yields a J_{sc} of 7.22 mA/cm², which is very close to 7.50 mA/cm² found for the photovoltaic devices confirming our hypothesis about the origin of the initial short circuit current discrepancy.

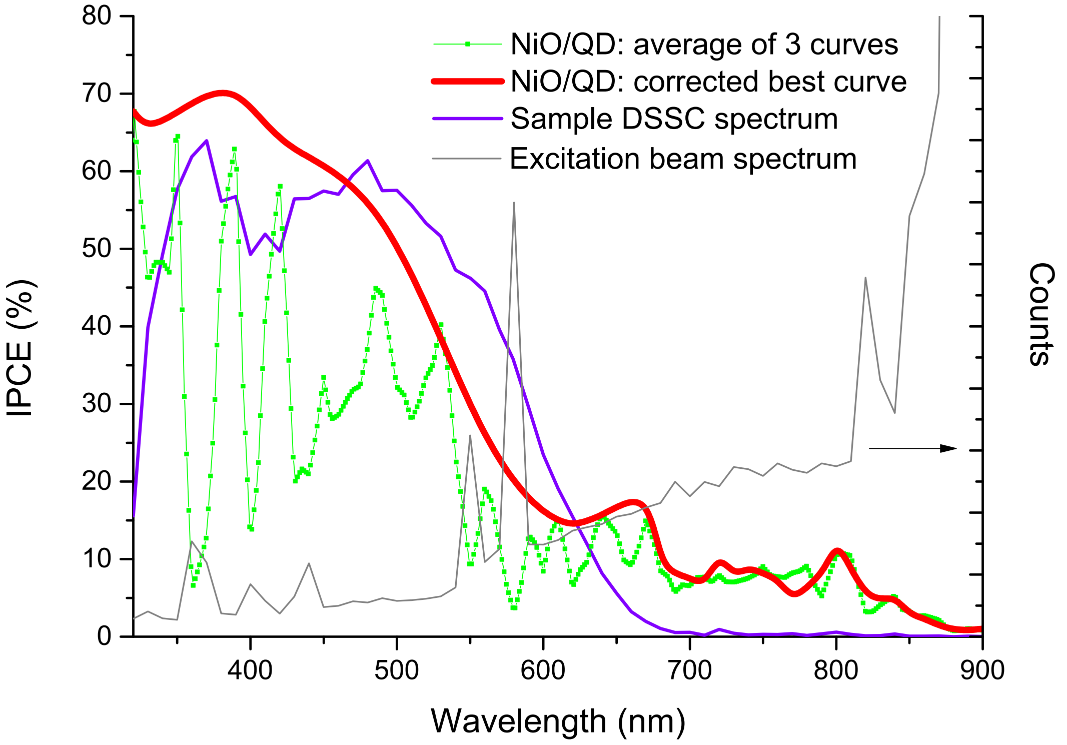

Figure S12. Green: IPCE spectrum of NiO/$CuInS_XSe_{2-x}$:Zn^{2+} QDs (average for 3 different solar cells); Red: IPCE spectrum corrected for the source contribution. Blue: spectrum for a sample DSSC using standard configuration; Grey: Hg-Xe excitation beam spectrum shown for comparison.

S1. T. Prill, K. Schladitz, D. Jeulin, M.Faesell and C. Wieser, *J. Microsc.*, **2013**, *250*, 77-87.

S2. M. C. Biesinger, B. P. Payne, L. W. M. Lau, A. Gerson, R. S. C. Smart, *Surf. Interface Anal.* **2009**, *41*, 324.

S3. S. L. Castro, S. G. Bailey, R. P. Raffaelle, K. K. Banger, A. F. Hepp, *J. Phys. Chem. B* **2004,** *108*, 12429-12435.

S4. B. Chen, H. Zhong, W. Zhang, Z. a. Tan, Li, Y.; C. Yu, T. Zhai, Y. Bando, S. Yang, B. Zou, *Adv. Funct. Mater.* **2012,** *22*, 2081-2088.

S5. Ward, A. J.; Ruseckas, A.; Samuel, I. D. W. *J. Phys. Chem. C* **2012,** *116*, 23931-23937.

S6. X. Z. Guo, Y. H. Luo, Y. D. Zhang, X. C. Huang, D. M. Li, Q. B. Meng, *Rev. Sci. Instrum.* **2010**, *81*, 103106.
